# Cultivation conditions and the diffusion of oxygen into culture media: The rationale for the flask-to-medium ratio in microbiology

**DOI:** 10.1186/1471-2180-13-9

**Published:** 2013-01-16

**Authors:** Greg A Somerville, Richard A Proctor

**Affiliations:** 1 School of Veterinary Medicine and Biomedical Sciences, University of Nebraska, Lincoln, NE 68583-0905, USA; 2 Departments of Medicine and Medical Microbiology/Immunology, University of Wisconsin School of Medicine and Public Health, Madison, WI, USA

**Keywords:** Cultivation conditions, Aeration, Oxygen diffusion

## Abstract

Bacterial cultivation requires consideration of three things: The bacterial strain, cultivation medium, and cultivation conditions. Most microbiologists dutifully report their choice of strains and cultivation media in manuscripts; however, these same microbiologists often overlook reporting cultivation conditions. Without this information, it is difficult to determine if cultures were grown aerobically, microaerobically, or anaerobically. To cultivate bacteria aerobically, it is necessary to understand that oxygen does not readily diffuse into culture media; it needs help to get in. Microbiologists can do this by altering the flask-to-medium ratio, rpm of agitation, and/or the concentration of atmospheric oxygen, or by using baffled flasks.

## 

In 1861, Louis Pasteur observed that yeast cultivated under aerobic growth conditions had an increased biomass relative to yeast grown under anaerobic conditions, and that this increase in biomass correlated with a decrease in fermentative metabolism [1]. This observation would become known as the Pasteur Effect; however, it would take nearly a century to provide the metabolic explanations for this observation (*e.g.*, NAD^+^-dependent activation of isocitrate dehydrogenase and feedback inhibition of phosphofructokinase; [2]). During the century it took to arrive at the metabolic explanations of the Pasteur Effect, it was observed that this effect was not unique to yeast, but it was also observed in mammalian tissues [3] and bacteria [4]. Fundamental to all of these observations, were the cultivation conditions; specifically, the dissolved oxygen content of the culture media. To understand the effect of aeration on yeast, eukaryotic cells, bacteria, *etc.*, it is essential to have a basal level of knowledge about the diffusion of oxygen into water.

The flux of oxygen into water follows Fick’s first law; hence, it is significantly influenced by the diffusion coefficient. The diffusion coefficient for oxygen into water is 2.1×10^-5^ cm^2^/second at 25°C, while the diffusion coefficient for oxygen into air is approximately 0.2 cm^2^/second [5]. In other words, oxygen is nearly 10,000 times more diffusive into air than it is into water. One obvious reason for this difference between the diffusivity of oxygen into air versus water is the viscosity of water is 1.002 centipoise at 20°C while the viscosity of air is approximately 0.18 centipoise. If the concentration of oxygen or the pressure is increased, then the flux of oxygen into water will increase even though the viscosity of water remains constant. From a biological perspective, it is uncommon to use saturating oxygen concentrations or high pressures. Hence, most biological experiments rely on an oxygen concentration of 20.946%; specifically, the concentration of oxygen in dry air at sea level and at 25°C. What does all of this mean to a biologist? The dissolved oxygen content of distilled water at 25°C is 5.77 ml/L [6], a concentration insufficient to support most aerobic life forms that do not have gills. This gets more complicated when we take into account that cultivation of biological specimens is never performed in pure water, but in water having dissolved solutes (*e.g.*, electrolytes and metabolites). A 10% solution of sodium chloride at 25°C has a dissolved oxygen content 5.21 ml/L [6], so the more concentrated the cultivation medium, the less oxygen is available to the biological specimens. In addition, it is common to autoclave culture media at 121°C at 15 psi of pressure for 15 minutes to sterilize the media. Heating water to a temperature of 100°C results in the deaeration of water. While the pressure in the autoclave is maintained, deaeration is reduced; however, once the pressure is lost and the media are still boiling deaeration will occur.

As stated, the diffusion coefficient for oxygen into water is very small, resulting in a minimal depth of penetration into culture media [7]. In a static culture, the diffusion of oxygen into the medium only occurs at the very top of the surface of the liquid that is exposed to the atmosphere; hence, everything below about 1 mm is growing anaerobically. To overcome a lack of oxygen in the media, the easiest solution is to increase the surface area exposed to the atmosphere. The two more commonly used methods for increasing the surface area are: decreasing the volume of media contained in a flask, such that there is only a thin layer of culture medium in the flask; and agitating the culture, such that the media is forced into thin films. Typically, these two methods are combined such that the volume of medium contained in a culture flask will form a thin film when agitated on a rotary shaker due the application of centripetal force [8]. The formation of thin films of culture media can be aided by the use of baffled flasks, which create bubbles and increase the surface area exposed to the atmosphere [9]. Taken together, aeration in batch cultures is a function of the volume of culture media in the flask, agitation speed, and the use of baffled flasks. In practice, the flask-to-media ratio, rpm of aeration, and the use of baffled flasks must be empirically determined for the task at hand and the biological specimen being cultured.

Cultivation conditions that influence the diffusion of oxygen into culture media will alter metabolism, electron transport, redox poise, *etc.*, causing regulatory changes (*e.g.*, [10]) that will alter the synthesis of bioproducts. For these reasons, it is important to carefully consider the cultivation conditions when designing an experiment. As an example, changing the flask-to medium ratio from 7:1 to 4:1, with 160 rpm of agitation, causes *Staphylococcus epidermidis* to transition from producing acetic acid to producing lactic acid when grown in tryptic soy broth containing glucose, a change that coincides with an increase in the accumulation of polysaccharide intercellular adhesion, the extracellular matrix of a biofilm [11]. As illustrated in this example, it is imperative that authors accurately report, and editors demand, the reporting of specific cultivation conditions [12].
